# An analysis of clinical application effects of 3d-printed act titanium trabecular intervertebral fusion cage in posterior lumbar interbody fusion (PLIF)

**DOI:** 10.12669/pjms.40.8.8759

**Published:** 2024-09

**Authors:** Zhijun Li, Hong Wang, Zhaoqi Wang, Zhiming Qi, Qi Yao

**Affiliations:** 1 Zhijun Li, School of Graduates, Dalian Medical University, Dalian, 116044, Liaoning, China; Department of Orthopedics, The Second People’s Hospital of Dalian, Dalian, 116011, Liaoning, China; 2 Hong Wang, Department of Orthopedics, Dalian Municipal Central Hospital Affiliated of Dalian Medical University, Dalian 116011, Liaoning, China; 3 Zhaoqi Wang, Department of Physiatry, The Second People’s Hospital of Dalian, Dalian 116011, Liaoning, China; 4 Zhiming Qi, Department of Orthopedics, The Second People’s Hospital of Dalian, Dalian 116011, Liaoning, China; 5 Qi Yao² Department of Orthopedics, The Second People’s Hospital of Dalian, Dalian 116011, Liaoning, China

**Keywords:** 3D printing, titanium trabecula, intervertebral fusion cage, Posterior lumbar interbody fusion (PLIF), Polyetheretherketone (PEEK)

## Abstract

**Objective:**

To compare the clinical effects of applying a 3D-printed ACT titanium trabecular intervertebral fusion cage and a Polyetheretherketone (PEEK) cage in posterior lumbar interbody fusion (PLIF).

**Methods:**

This was a clinical comparative study. Forty patients with degenerative lumbar diseases admitted at The Second People’s Hospital of Dalian from January 2020 to December 2021 were selected and divided into an observation group (3D cage) and a control group (PEEK cage) using the random number table method, with each group of 20 cases. The visual analogue scale (VAS) scores, Japanese Orthopaedic Association (JOA) scores, Cobb angles at fusion segments, intervertebral height and intervertebral fusion situations of the patients between the groups were compared.

**Results:**

No significant differences were found in their operation time, intraoperative blood losses and operation related complications(p>0.05). In terms of postoperative VAS and JOA scores in both groups, they are all significantly improved compared with those before the operation, and their differences are also statistically significant(*p<*0.05). However, no statistical significance exists in inter-group differences(p>0.05). Postoperative Cobb angles and intervertebral height of patients in both groups are considerably bettered compared with those before the operation. Their differences show statistical significance(*p<*0.05), while inter-group differences are proved to be not statistically significant(p>0.05).

**Conclusions:**

Applying a 3D-printed ACT titanium trabecular intervertebral fusion cage or PEEK cage in PLIF has the potential to improve clinical symptoms of patients with degenerative lumbar diseases, and restore the Cobb angle and intervertebral height. 3D-printed ACT titanium trabecular intervertebral fusion cage can accelerate intervertebral fusion without increasing operation related complications.

## INTRODUCTION

With lifestyle changing and population aging, degenerative lumbar diseases have become increasingly common and frequently seen in the orthopedics department, hindering patients’ daily life and work.^1^,^2^ Surgery is considered an effective treatment in the event of conservative treatment failure. Posterior lumbar interbody fusion (PLIF), typical in treatment of degenerative lumbar diseases, aims to relieve neurothlipsis and restore intervertebral height and physiological curvature of lumbar.^3^,^4^ In early phases, PLIF has a rather high failure rate and complication incidence. Thanks to the invention and application of interbody fusion cages, the fusion rate of PLIF has been significantly improved, while complications are reduced accordingly. Now, interbody fusion cages made of different materials and structures have been clinically used.^5^,^6^ Among them, Polyether ether ketone (PEEK) Cage is the most common one. Elasticity modulus of the PEEK cage is similar to that of bone tissues, and stability and biocompatibility of such a cage are both preferable. In spite of this, PEEK cage still has a rather prominent flaw of difficulty in binding to bone tissues, which leads to slow fusion. In later phases, it is much likely for settlement and other problems to occur.^7^ In recent years, with the rapid progress of 3D printing technologies, 3D-printed porous ACT titanium trabecular cages have been used in clinics. These cages feature a large contact area, low stress shielding, high biocompatibility and an elasticity modulus close to that of the vertebral body.^8^

However, their clinical application is short in time^9^ and mostly in cervical vertebra. As for its clinical safety and validity of being applied in lumbar vertebra, reports are rarely seen, thus lacking clinical evidences of evidence-based medicine. As such this study was conducted to comparatively analyze effects of the 3D-printed ACT titanium trabecular cage and the PEEK cage in PLIF, aiming to provide evidences of evidence-based medicine for clinical applications of 3D-printed ACT titanium trabecular cages.^10^

## METHODS

This was a clinical comparative study. According to the following inclusion and exclusion criteria, 40 patients with degenerative lumbar diseases received at The Second People’s Hospital of Dalian from January 2020 to December 2021 were selected as the research object using the random number table method divided into observation group (3D cage) and control group (PEEK cage), with each group of 20 cases.

### Ethical Approval:

The study was approved by the Institutional Ethics Committee of The Second People’s Hospital of Dalian (No.: 20200110; date: January 10, 2020), and written informed consent was obtained from all participants.

### Inclusion criteria:

- Age: 18-75;
- Patients, conforming to diagnostic standards of lumbar spinal stenosis, prolapse of lumbar intervertebral disc, and lumbar spondylolisthesis, who have no responses to half-year conservative treatment and plan to receive PLIF;
- Patients with single segmental lesions;
- Patients with complete and valid peri-operative data;
- Patients signed the informed consent.

### Exclusion criteria:

- Patients confirmed or suspected to be allergic to implant;
- Patients with spinal tumors or infectious diseases;
- Patients with serious systemic diseases and intolerant of surgeries;
- Patients with peri-operative data incomplete.
- Patients with coronary heart disease, diabetes and other basic diseases.

After the participants were discharged from the hospital, they were followed up until December 2022. Materials The 3D-printed ACT titanium trabecular cage was provided by AKMEDICAL. As an irregular polyhedral, this cage has solid edges, porous end faces, slots and screw holes at the tail end, and an arc-shaped head end. PEEK Cage was manufactured in Wego Ortho Materials Co., Ltd. (Shandong, China).

### Surgical methods:

All operations were performed by the same surgical team. After general anesthesia, the patients lay prostrate on an orthopedic operating table. Fluoroscopy of the C-arm was implemented to identify and mark operative segments. Posterior median incisions were made on the lumbar regions. Through incision layer by layer, spinous processes, vertebral plates, and articular processes were all exposed. Based on the screw fixation angle established before the operation, pedicle screws were implanted. Since fluoroscopic pedicle screw positions satisfy relevant requirements, upper and lower vertebral plates, spinous processes, and articular processes of the target joint space were excised to fully expose endorhachis and nerve roots. Thus, pre-bending connecting rods of proper length can be implanted to complete intervertebral space distraction, target disc excision, and cartilage endplate curettage. After that, 3D or PEEK Cages of an appropriate model number were placed. Both dura cysts and nerve roots should be examined to again ensure no compression. Finally, a fluoroscopy of C-arm was performed to confirm that both the cage and the rod system have been accurately positioned and solidly fixed. In this case, drainage tubes were placed and the incision sewed up layer by layer. All patients were treated with cage by strictly trained personnel, and the researchers were proficient in both fixation methods.

### Postoperative treatment:

After the operation, drugs should be administered for anti-infection and pain relief. If the drainage volume was below 50 mL in 24 hour after the operation, the drainage tube should be removed. For incisions, dressings should be changed regularly and stitches be taken out. Within three months after the operation, the patient should wear a waist support move around, and within six months after the operation, strenuous physical exertion is prohibited. Two surgeons are respectively responsible for data statistics about and follow-up visits in three, six, and twelve months after the operation.

### Observation indicators:

The operation time, intra-operative bleeding volumes and operation-related complications of both groups should be recorded. VAS scores were graded before the operation and in six and twelve months after the operation to grade the pain of patients. More particularly, ten points represent sharp pain, while zero stands for no pain. In addition, JOA scores were also adopted to evaluate patients’ lumbar vertebral functions (full score of 29, with the lowest of 0). The higher the score is, the more significant improvements in lumbar vertebral functions will be. Six and twelve months after the operation, X-ray films were taken to measure Cobb angles of fusion segments as well as the intervertebral height, i.e., the distance from the midpoint of inferior endplate tangent of the upper vertebra to that of superior endplate tangent of lower vertebra. At three, six and twelve months after the operation, fusion conditions should be evaluated. By virtue of CT images, intervertebral fusion was graded into I-V levels according to evaluation standards proposed by Brantigan and Steffee. During the last follow-up visit, effective rates of fusion were obtained from patients in both groups.

### Statistical analyses:

SPSS21.0 was selected to make statistical analyses. Relevant measurement data were denoted by Mean±Standard Deviation (*χ̅*±*S*), while enumeration data by the number of cases and their percentage [n (%)]. Data in this study were analyzed using a 95% confidence interval. To perform inter-group comparisons, independent samples t-tests were carried out, and c^2^ tests or Fisher’s exact tests were conducted to verify inter-group comparison results. Inter-group comparison of ranked data was fulfilled by rank sum tests. In the event of *p<*0.05, differences are considered statistically significant.

## RESULTS

The observation group included 20 patients (eight males and twelve females) aged 36-60 (mean age: 52.50±7.51). There were 20 cases in the control group, including 10 males and 10 females aged 28-60 (mean age: 50.65±9.43). General data of patients in both groups show no significant differences (p>0.05), but are comparable. For details. Table-I

**Table-I:** General data comparison for patients in observation and control groups.

Items	Observation group (n=20)	Control group (n=20)	t/c^2^	P
Age (*χ̅*±*S*)	52.50±7.51	50.65±9.43	0.686	0.497
Sex (n, M/F)	8/12	10/10	0.525	0.751
BMI (*χ̅*±*S*, kg/m^2^)	33.30±3.15	33.05±2.63	0.273	0.786
Medical history (n)			0.428	0.807
Lumbar spinal stenosis	8 (40.00)	7 (35.00)		
Protrusion of lumbar intervertebral disc	7 (35.00)	9 (45.00)		
Lumbar spondylolisthesis	5 (25.00)	4 (20.00)		
Operative segments (n)			0.178	0.915
L3/4	5 (25.00)	4 (20.00)		
L4/5	8 (40.00)	8 (40.00)		
L5/S1	7 (35.00)	8 (40.00)		

Surgeries were all smoothly completed for both groups. Regarding their operation time, intra-operative bleeding volumes, and operation related complications, no significant differences are found, p>0.05.Table-II.

**Table-II:** Comparison of operation-related information between observation and control groups.

Items	Observation group (n=20)	Control group (n=20)	t/χ^2^	P
Operation time (*χ̅*±*S*, min)	154.75±6.17	152.80±8.76	0.814	0.421
Bleeding volume (*χ̅*±*S*, mL)	343.25±7.30	342.75±8.35	0.202	0.841
Complications (n)			2.917	0.233
Leakage of cerebrospinal fluid	1 (40.00)	0 (35.00)		
Incision infection	0 (35.00)	2 (45.00)		
Urinary tract infection	1 (35.00)	1 (40.00)		

VAS and JOA scores after the operation are all significantly improved than those before the operation in both groups, with statistically significant difference (P< 0.05). But inter-group comparative differences show no statistical significance (p>0.05), as presented in Table-III.

**Table-III:** Pre-operative and post-operative VAS/JOA score comparison in observation and control groups (*χ̅*±*S*).

Groups	Time	VAS scores (points)	JOA scores (points)
Observation	Pre-operative	8.05±0.83	7.70±0.73
	6 months after operation	2.25±0.44^ab^	20.65±0.67^ab^
	12 months after operation	0.55±0.51^ab^	25.20±1.11^ab^
Control	Pre-operative	7.90±0.72	8.00±1.03
	6 months after operation	2.40±0.50^a^	20.20±1.61^a^
	12 months after operation	0.60±.050^a^	25.70±1.13^a^

***Notes:*** (1)

a P<0.05, if compared with the pre-operative data of the same group; and (2) ^b^p>0.05 in contemporary comparison with the control group.

Post-operative Cobb angles and intervertebral height in patients from both groups are all substantially improved if compared with those before the operation, with statistically significant differences, *p<*0.05. But no such significance exists in inter-group comparison, p>0.05, as shown in Table-IV.

**Table-IV:** Pre-operative and post-operative Cobb angle/intervertebral height comparison in observation and control groups (*χ̅*±*S*).

Groups	Time	Cobb angle (°)	Intervertebral height (mm)
Observation	Pre-operative	8.20±0.24	7.32±0.84
	6 months after operation	12.45±0.55^ab^	10.56±0.59^ab^
	12 months after operation	11.79±0.93^ab^	10.41±1.28^ab^
Control	Pre-operative	8.45±0.32	7.12±0.69
	6 months after operation	12.53±0.31^a^	10.91±0.93^a^
	12 months after operation	11.80±.1.32^a^	10.22±0.40^a^

***Notes:*** (1)

a P<0.05, if compared with the pre-operative data of the same group; and (2) ^b^p>0.05 in contemporary comparison with the control group.

At three and six months after the operation, intervertebral fusion classifications in the observation group were found superior to those in the control group. Their differences show statistical significance (p<0.05). At twelve months after the operation, inter-group differences in intervertebral fusion have no statistical significance (p>0.05). For details, please refer to the following Table-V.

**Table-V:** Comparison of post-operative intervertebral fusion classifications in observation and control groups (n).

Groups	3-month					6-month					12-month				

	I	II	III	IV	V	I	II	III	IV	V	I	II	III	IV	V
Observation	0	5	12	3	0	0	0	2	12	6	0	0	0	5	15
Control	0	13	6	1	0	0	2	10	6	2	0	0	1	6	13
Z-value	-3.024					-2.415					-0.334				
P-value	0.002					0.016					0.738				

## DISCUSSION

In this study, the 3D-printed cage was used together with a few autogenous bones. Although the model number of the 3D-printed cage selected before the operation can be roughly identified according to imageology, model testing was still carried out during the operation. The findings show that no statistical significances lie in comparative differences between the operation time and intra-operative bleeding volumes in both groups (*p>*0.05). Comparatively, some literature reported that a 3D-printed ACT titanium trabecular intervertebral fusion cage can be used to significantly shorten the operation time and reduce intra-operative bleeding volumes for the following reasons:

3D-printed cages may contribute to reducing the number of model tests and the frequency of implantation difficulty, while for PEEK cage, model testing should be performed repeatedly during implantation to select a proper cage, which extends the time of operation and causes more damages to tissues.^11^,^12^

The 3D-printed cage has a porous structure and does not need bone grafting, which the spares the time consumed by bone grafting.^13^,^14^

Posterior lumbar interbody fusion (PLIF) is performed to eliminate clinical symptoms induced by degenerative lumbar diseases and improve the life quality of patients. Under such a circumstance, VAS and JOA ratings are commonly used observation targets for evaluating the clinical effects of the spinal operation. According to our research results, postoperative VAS scores of patients from both groups are all considerably improved compared with those before the operation. Their comparative differences before and after the operation are statistically significant (*p<*0.05), while inter-group comparison shows no obvious differences (p>0.05). Likewise, JOA scores are also noticeably improved compared with those before the operation. Statistical significance exists in their differences before and after the operation (*p<*0.05), while inter-group comparison produces no significant differences (p>0.05). The results indicate both 3D-printed ACT titanium trabecular intervertebral fusion and PEEK cages can relieve pain caused by degenerative lumbar diseases and restore neurological function, and the fact of no significant differences in inter-group comparison results manifests that the 3D-printed cage can generate the same clinical effects as the PEEK cage does.

Lumbar interbody fusion has been extensively applied with its capability of effectively restoring the Cobb angle and raising the success rate of bone graft fusion. The level of Cobb angle restoration and fusion has become the major focus of spine surgeons^15^,^16^, as favorable Cobb angle restoration and rapid intervertebral fusion are conducive to the early rehabilitation exercise and recovery of the patients, thus ensuring satisfactory surgical effects and improving life quality of patients^17^. In this study, post-operative Cobb angles in patients from both groups are all significantly improved compared with those before the operation, and their differences before and after the operation also show statistical significance (*p<*0.05). While, no significant differences were found in the inter-group comparison (p>0.05), indicating that the 3D-printed ACT titanium trabecular intervertebral fusion cage can achieve the same Cobb angle restoration effect as the PEEK cage. In terms of PEEK cages, their fusion efficiency is lower than that of the 3D-printed ACT titanium trabecular intervertebral fusion cage due to the unique biological traits of the former.^18^ As for the 3D-printed cage, an oxidation layer on its surface has the potential to facilitate the binding of multiple extracellular matrix proteins to this surface, thus providing a good environment for osteoblast differentiation and adherence.^19^ Besides, the surface roughness and high porosity of the 3D-printed ACT titanium trabecular intervertebral fusion cage also provide space for adherence, proliferation, and differentiation of bone tissues, leading to faster intervertebral fusion.^20^ The findings show better fusion effects from the observation group three months after the operation than the control group (p<0.05). For half a year after the operation, most patients in the observation group show synostosis, while the proportion of patients with synostosis in the control group is lower than the observation group (p<0.05). One year after the operation, synostosis is observed in all patients from the observation group, p>0.05.

### Limitations of the study:

It includes a small sample size and a short period of follow-up visits. It failed to make a comparison with the parameters of the normal population, so further clinical observation and investigations should be conducted.

## CONCLUSIONS

Applying the 3D-printed ACT titanium trabecular intervertebral fusion cage in PLIF can effectively relieve patients’ clinical symptoms, facilitate neurological function recovery, restore the Cobb angle and the intervertebral height, produce a clinical effect equivalent to those of the commonly used PEEK cage, and realize a faster intervertebral fusion.

### Authors’ Contributions:

**ZL and** **HW** carried out the studies, data collection, drafted the manuscript, and are responsible accountable for the accuracy or integrity of the work.

**ZW and ZQ** performed the statistical analysis and participated in its design.

**QY** participated in acquisition, analysis, or interpretation of data and draft the manuscript.

All authors read and approved the final manuscript.
